# Theoretical Models of Collaborative Partnerships in Arts-Health Care Practices for Older Adults

**DOI:** 10.3390/ijerph20196888

**Published:** 2023-10-06

**Authors:** Dohee Lee, Masood Masoodian

**Affiliations:** School of Arts, Design and Architecture, Aalto University, 02150 Espoo, Finland; masood.masoodian@aalto.fi

**Keywords:** arts-health practices, arts-based interventions, cultural activities, co-creativity, co-production, interdisciplinary collaboration, older adults, practice framework

## Abstract

Although research investigating collaborative partnerships with older adults has been slow to develop, promoting user involvement and co-production is gaining interest in aging studies, with the aim of improving interactions between the different stakeholders involved, and toward the more effective delivery of care provisions and better community life for aging people. This is based on existing evidence that improved dynamics within collaborative and mutual learning processes can enhance the integration of new practices at different levels by generating novel creative approaches and practice frameworks for the delivery of quality care for older adults. This article presents the findings from a series of narrative interviews conducted with different stakeholders involved in *arts-health practices* in Finland and South Korea. Focusing on empirical perspectives of these stakeholders on arts-health practices—from planning to assessment—this study identifies vital components of co-producing and co-delivering arts-health practices for older adults and highlights the importance of utilizing their late-life creativity as active partners in such practices across cultural contexts. In addition to identifying three central stages of developing arts-health practices, two theocratical models are proposed to provide structural support for collaborative partnerships in arts-health practices, with the aim of promoting holistic care provisions for aging people through such practices.

## 1. Introduction

A modern society is organized through a diversity of progressive and specialized fields of thought, policy, and activity [1]. Health and social care policies in particular are increasingly following this trend in many societies by promoting user involvement in various aspects of planning and policy making [2,3,4]. Although users’ involvement in research processes, and the benefits of this involvement for research outcomes, have attracted considerable attention recently, not much is known about how individual users take on the role of lay researchers in their own communities [5].

This user involvement in co-research and co-production is becoming particularly important for developing health and social care policies for older adults, in part due to an increasingly aging population worldwide and the need for more inclusive and responsive policies and services in aging societies [6]. In addition, to address global issues such as agism, well-being, and quality of later life, health and social care services are under increasing pressure to improve the quality of services offered to older people [7]. This has led to a greater understanding that the quality care for older adults should go beyond merely meeting their basic physical healthcare needs and should also aim to improve their quality of life and general well-being [8]. These include older adults’ individual needs such as preserving their sense of identity, creating opportunities for them to participate in social activities, maintaining their emotional and spiritual well-being, amongst others.

One area of increasing interest in that direction is to provide arts-based interventions and cultural activities for aging people, as an integral part of improving their creative well-being in later life [9]. To achieve this, however, it is necessary to pursue a shared vision and foster a broad range of social integration among multiple stakeholders who are striving to meet mutual public health goals—including older adults themselves and the health and social care sectors. There are also many other associated collaborators involved in providing arts-based interventions and cultural activities for older adults, including, for instance, artists and facilitators, arts and cultural organizations, community organizations, local and national government agencies, and so on. To facilitate collaboration between these multiple stakeholders from across diverse fields—sometimes with diverging interests and concerns—novel creative approaches and practice frameworks are needed for providing quality health and social care for older adults. These approaches and frameworks must also be able to bridge unsettled interdisciplinary relationships among a wide range of fields involved, such as health and well-being, arts and aesthetics, community and cultural development, social policy and social justice, and local and national politics, to name a few.

This article presents a study based on a series of narrative interviews conducted in Finland and South Korea between 2019 and 2022. This study focused on these two countries due to their rapidly aging populations posing challenges to their policy-makers and fiscal institutions, as well as a mounting economic burden on the working-age populations [10,11]. As a result of such demographic changes in both countries, their social welfare and health care systems are being reformed with the aim of reducing health disparities and improving equality and accessibility toward social cohesion, as well as promoting the active participation of older adults in society [10,11]. The choice of two similarly industrialized countries with presumably different cultural settings—European and Asian—is also meant to highlight any major differences that might exist between them in their approaches to addressing the social and health care needs of their aging populations due to their different cultural contexts.

The objective of this study has been to draw out the empirical perspectives of different stakeholders—including older adults, practitioners, and agencies—involved in a broad range of *arts-health practices* for aging people, mapping out their relationships as collaborators, as well as highlighting the growing importance of older adults themselves in becoming valuable co-investigators and active partners in *arts-health practices*. In this context, the term *arts-health practices* is used here to refer to a wide range of arts-based interventions and cultural activities targeted at older adults, with the aim of contributing to their creative well-being as part of their healthy aging [9]. Therefore, *arts-health practices* encompass not only creative activities undertaken in institutional care settings—such as artistic activities carried out in day-care centers and residential homes for older adults—but also other everyday creative activities carried out as part of the daily lives of older adults—such as enjoying arts, making arts and crafts, visiting museums and art festivals, and so on. 

The study presented here examines arts-health practices from the planning to assessment stages, through a multi-layered investigation across cultural contexts, to identify the vital components of designing arts-health practices for older adults’ *creative well-being*—referring to creativity as a source of well-being in general, and a source of physical, mental, and social well-being in particular [8,9]. This study also aims to highlight older adults’ *late-life creativity* that intimately intertwines their strength and challenges, encouraging them to share the power of decision-making with other stakeholders involved in arts-health practices for them. In addition, this research explores ways of creating a cyclic structure for operating arts-health practices in an interdisciplinary environment that distributes responsibilities and opens decision-making processes across different stakeholders. Finally, based on the outcome of this study, this article proposes two theoretical models aimed at promoting holistic care provisions in arts-health practices through collaborative partnerships between multiple stakeholders involved in such practices.

## 2. Changing Paradigm in Care for Older Adults

Leadbeater [12], in “Personalisation through participation: A new script for public services”, examines the concept of “*personalisation*” in public services, and emphasizes a “deep” personalization as a more radical and disruptive innovation approach, rather than a “shallow” one (p. 20). This kind of deep personalization aims to allow users to actively participate in the design and provision of services for them, as co-designers and co-producers with a much larger role and responsibility, rather than just dependent consumers [12]. Along with the changing users’ role to include increased participation, commitment, knowledge, and responsibility, Leadbeater [12] also argues for a change in the role of professionals to include ways of creating platforms, peer-to-peer support networks, and environments where people can work together to find solutions collaboratively. With these changes, different public services such as community safety initiatives, older adults’ rehabilitation care programs, and many welfare-to-work programs can be redesigned to be more adaptive and, by doing so, prevent users from becoming dependent on the government, and therefore even lead to the emergence of an entirely new organizational approach for arranging social services [12].

Here, we use this deep personalization approach to focus on the design and delivery of arts-health practices involving older adults. We start by reviewing the concept of user involvement and co-production that has emerged as a growing area of interest in the field of aging studies, that has resulted in greater interaction between stakeholders, and that has led to improved care provisions and community life for older adults. Based on this, we then explore how older adults’ late-life creativity can be utilized to empower them to contribute to value production and become more capable of participating in decision-making processes in arts-health practices.

### 2.1. Co-Production: Older Adults as Co-Researchers

Over the past three decades, the involvement of users in the design and delivery of services has become more mainstream [13]. In terms of the depth and scope of such involvement, it can range from being merely superficial—just decorative—to being more integral, and therefore more effective [14]. The concept of “*co-production*” is particularly relevant here, being grounded in the idea of partnership and equality; it aims to empower and make preciously excluded groups valuable partners [15]. As part of this approach, “*person-centered planning*” can be very effective in serving as a catalyst, encouraging a genuine partnership between the professionals and those people they want to help [15].

Co-production in the public service domain is often linked to two main perspectives: public administration and service management [16]. Among the various modes of co-production defined by Osborne and Strokosch [16], “*enhanced co-production*” integrates both perspectives, by combining co-production in strategic planning with the operational management of public services (p. S37). This mode proposes new forms of public service delivery called “*user-led innovation*”, which challenge the entire public service delivery, and aim to even potentially lead to transformational innovation in the service paradigm by explicitly emphasizing the role of service users as a driving force [16] (p. S39). By doing so, enhanced co-production can affect the behavior of the relevant stakeholders—including service users and facilitators—when designing and orchestrating diverse interrelated domains, through integration of new practices at all levels of individual, organizational, and systemic [17].

In a similar vein to user involvement in enhanced co-production, there is a need to involve older adults in aging research as co-researchers. It is increasingly being realized that partnering with users in research can help identify research priorities, formulate research questions, and generate credible results [18]. While research in partnership with older adults has been slower to develop than research with other user groups (e.g., see [6,19]), in the field of aging studies, older adults have in some cases been involved as co-researchers at various stages of research. Bindels et al. [20] have, for instance, noted that “aging research finds itself in a new situation, with a top-down trend towards consumerism, increased user involvement required by funding agencies and a bottom-up surge of social movements comprised of older people who desire increased control over the decisions which affect their lives” (p. 2). This indicates that there is a notable trend in aging research in which older adults are receiving well-deserved attention as experts, and through their experiential knowledge, they are now able to influence research as a whole, “from research agenda setting and research design to research evaluation and dissemination of research result” [21] (p. 161). The active involvement of older adults in research as co-researchers also provides them with a sense of purpose and satisfaction, by allowing them to make positive contributions to research, while at the same time empowering them and increasing their knowledge, skills, and self-confidence [22].

Despite these developments, however, different competencies of older adults are often underestimated due to widespread stereotypes and negative public conceptions about aging [23], which can make older people less likely to participate actively in research as co-researchers [24]. It is, therefore, important to recognize that older adults have a unique perspective on aging and care services, due to their own accumulated lived experiences of aging and the care services they receive [20]. Since partnership entails all stakeholders working together to identify what needs to be done and how to accomplish it most effectively [24], understanding subjective experiences of aging can be considered as a foundation for providing more appropriate support mechanisms and services to older adults [4]. In addition, by recognizing the agency of older adults in co-planning their own care, researchers can motivate older adults and their caregivers to take on meaningful roles in research by, for example, collaborating as advisors, interviewers, co-researchers, or even initiators of research [20,21].

Overall, research agendas and methods in aging studies must be tailored to fully engage older adults at all stages of the research process. By doing so, older adults can be legitimized as active clients, and thinking individuals with the right to be themselves, thus going beyond merely being just research participants. When this legitimacy eventually leads older adults to describe themselves in terms of agency, they can often eliminate age-related terms such as frailty, decline, and disability [25]. As a result, agism and the pervasive “deficit” view of aging can be reframed into something resourceful and respectful. This changing view can in turn help older adults pursue “a much more positive ethos of rights, participation, empowerment, and interdependency” rather than being dependent and cared for [24] (p. 66). 

### 2.2. Empowerment through Late-Life Creativity

In general, empowerment involves enabling people to recognize their strengths so that they can develop power and gain control over their lives [26,27,28,29,30,31,32]. Due to its importance, empowerment has been embedded into many other concepts such as “*corporate social responsibility (CSR)*” [33,34], “*governmentality*” [35], and “*responsibilization*” [36], all of which aim to lead to responsible actions that are derived from self-regulated and collaborative efforts. Similarly, empowerment can also be related to late-life creativity [8].

According to Lindauer [37], late-life creativity is shown by “changing occupations, careers, and professions, or starting a new one; setting new goals or redirecting old ones; moving on to new ways of thinking and modes of imagination; posing new questions and pursuing unfamiliar lines of inquiry; searching within oneself for submerged talents and recharging hidden interests; and behaving in ways that were not just different but better than earlier efforts” (p. vii). In other words, late-life creativity is an important “source of self-discovery and self-creation” [38] (p. 3), as well as a means of achieving greater self-confidence and maturity as people age [38,39,40,41,42]. Therefore, late-life creativity can also be an effective way of focusing on the positive aspects of aging and encouraging older people to cope with the aging process itself [43,44], not to mention acting as a powerful tool that provides transcendence, wisdom, well-being, and self-fulfillment in later life and offers new insights into aging [42].

The conventional views of late-life creativity often fail to consider the sociocultural and relational components of creativity, and their connections with lived experiences [42]. In addition, the existing theories about the underlying mechanisms of creativity—attributing it to anything from a method to madness—are not really satisfactory [45]. Nonetheless, according to Formosa [46], there are considerable overlaps between empowerment and creativity, because “[a]daptability, flexibility, and coping are all inherent processes in the creative activity” (p. 84), just as they are part of empowerment. The reason for this is that creativity provides individuals with the ability to develop new ideas and approaches when solving a problem or challenge [46]. In particular, the physiological and psychosocial benefits gained from older adults pursuing creative activities lead to empowering them and improving their active and productive aging [46]. Similarly, Cohen [47] examines the importance of a theoretical approach in terms of creativity and aging, focusing on a series of human developmental phases in the second half of life. Cohen [47,48] emphasizes that individuals’ dynamics of inner drives foster their psychological growth throughout their life cycle, and such inner drives can inspire older adults’ untapped capacity and stimulate their creative potential as they age. This allows older adults to adapt and shape any tools and resources they have to express a vision or idea using their accumulated skills [49]. 

These theories have in recent years been substantiated by a growing body of evidence-based investigation (e.g., see [43]) and empirical research relating to late-life creativity and older adults’ creative engagement—conducted in gerontological studies, and social and psychological sciences—and accompanied by further theoretical developments (e.g., see [50,51]). For instance, in a study involving critical analysis of late-life creativity—conducted within the context of sociology in valuation—Gallistl [52] suggests that the “change in perspective on late-life creativity consequently sheds new light on the value older adults’ experience through their creative engagement” (p. 2600) and notes that late-life creativity is “a process of value production” (p. 2610). This means that the concept of late-life creativity does not just refer to older adults’ engagement in society but also to a more ubiquitous norm and expectation that older adults can become part of a later-life entrepreneurial culture [52]. 

In summary, the power of late-life creativity can, therefore, be utilized to enhance individuals’ creative expression, improve their quality of life, and serve as psychological capital to benefit people’s mental and physical well-being as they age [53].

## 3. Study of Arts-Health Practices

The objective of this study was to examine the empirical perspectives of different stakeholders involved in a wide range of arts-health practices for aging people, as well as to explore their relationships as collaborators. The stakeholders involved in such practices include not only the older adults themselves but also different kinds of art practitioners and caregivers, as well as various other agencies. Through this empirical investigation across different cultural contexts, we attempted to identify the essential components necessary for designing arts-health practices for older adults to support their late-life creative well-being. This study also aimed to find effective methods of involving older adults as valued co-designers and co-creators, participating actively as decision-makers in arts-health practices together with other relevant stakeholders. 

Older adults are typically considered to be people aged 65 and over [54]—with 65 being the usual retirement age in many developed countries over the past few decades. Existing aging studies, however, classify older adults differently, with some defining older adults as those aged 56 and over (e.g., see [55,56]). Several studies use a more precise classification of older adults. For example, Alterovitz and Mendelsohn [56] have referred to older adults aged 60–74 as “young-old” and those over 75 as “old-old” (p. 160). In our study, we used a broader age range for what we consider as older adults, and therefore, our older adult interviewees fell between the ages of 58 and 86 years old.

### 3.1. Methodology

This study was primarily based on a series of narrative interviews conducted in Finland and South Korea between 2019 and 2022, in addition to reviewing several existing reports of arts-health practices. The study interviewees were either older adult participants or practitioners and facilitators who had taken part or worked in a broad range of arts-health practices. The interviews focused on gathering personal experiences of the interviewees in addressing the challenges related to such practices, and examining the empirical perspectives of different stakeholders—older adults, practitioners, and agencies—to identify the common elements needed across different arts-health practices, from their planning to evaluation stages. Table 1 provides a summary overview of the interview and report data sources used for this study. The data collection was multi-layered and included data collected through (a) interviews, (b) focus groups, and (c) program reports.

The individual interview data consisted of two sets of interviews organized with older adults, and practitioners and facilitators from different agencies. For the first set (I1), 9 individual interviews were conducted with older adults in Finland, the primary purpose of which was to gain a better understanding of their aging experience, as well as their needs for arts-health practices supporting their creative well-being. For the second set (I2), 15 individual interviews were conducted with practitioners and facilitators in Finland and South Korea. These interviews aimed to better understand the current state of art-health practices in the two countries and investigate their contributions to the creative well-being of older adults.

The focus groups were held with two different groups of older adults who were participating in specific arts-health practices specially designed for them. The two groups consisted of 9 older adults in Finland (F1) and 10 in South Korea (F2). The focus groups aimed to investigate the experiences of older adults in participating in arts-health practices and the value of such artistic engagements for their creative well-being. The focus groups were facilitated following a method proposed by Rubin and Babbie [57], in which “a small group of people are brought together to engage in a guided discussion of a specified topic” (p. 621). This method gathers not only the participants’ experiences, both at an individual and group level, but also provides an opportunity for the interviewer to discover more about the participants’ shared knowledge, while eliciting their forgotten stories and other valuable details, particularly about specific cases. 

Both the individual interviews and the focus groups followed the narrative inquiry method [58] in the form of semi-structured interviews with open-ended questions about the interviewees’ holistic experiences of the arts-health practices in which they had previously participated. While the word “narrative” refers to a “story”, in the context of narrative inquiry, narrative means something more than a story, by implying that the story has a more serious meaning and purpose [59] (p. 77). The narrative inquiry method has in recent years become a valuable qualitative method for empirical research in social sciences, due to the fact that it allows for capturing the interviewees’ perspective sequentially over longer periods of time [60]. Using this method, the interviewees are able to narrate their everyday lives—in their entirety or some interesting parts—to the researchers [60]. By retracing the interviewees’ personal life experiences and memories, the narrative interview method thus enables the participants to share their deliberate opinions beyond simplistic responses to interview questions. 

The narrative interviews were conducted using thematically categorized open-ended questions, and example keyword prompts were provided when necessary to evoke the interviewees’ memories in the retrospective dimension. Most of the interviews were conducted online due to the COVID-19 pandemic restrictions between 2020 and 2021, with a few of them conducted in person or in written form. The interviews took around 60–90 min and were conducted in a common language between the interviewer—the first author of this article—and the participants, either in Korean or English. A professional interpreter took part in the focus group held in Finland (F1) to translate between Finnish and English. In addition, to minimize any issues related to language differences, the facilitator of this group attended the focus group session to verify and confirm any narratives that might have been overlooked. All the individual interviews and focus group sessions were audio-recorded and transcribed by the interviewer—i.e., the first author. The interview data were anonymized to protect the interviewees’ identities and their confidential information. 

Lastly, the four final reports (R1) were shared by practitioners and facilitators who had taken part in different arts-health practices. Each of the final reports analyzed feedback received from the participants, practitioners, and facilitators of their individual arts-health practices, as well as the qualitative and quantitative evaluation of their specific practices. The final reports also included additional observations and comments from external professionals about the practices they had reviewed.

### 3.2. Data Analysis

The transcribed narrative interview data and field notes, as well as the final reports, were analyzed following a conventional content analysis method [61]. Using content analysis to make replicable and valid inferences from data to their context provides knowledge, new insights, a representation of facts, and practical information [62,63]. The goal of this method is to develop a broad and concise description of the phenomenon, which is then formalized in categories or concepts [62]. Those categories or concepts are often used to form models, conceptual systems, conceptual maps, or sets of categories [62]. 

Table 2 provides a summary of the categories identified in our study data using the content analysis method in an inductive way. Following an open coding process, and creating categories and abstraction [62], we identified and classified the key elements necessary for operating arts-health practices into three main categories, each related to one of the three central stages of organizing arts-health practices. The inductive approach that was followed moves from the specific to the general, by observing particular cases, and then summarizing them into larger wholes or generalizations [62,64]. Although the two countries included in this study had unique cultural differences in terms of their different political, health care and social welfare systems, and their bureaucratic procedures based on national characteristics, the thematically categorized interview questions and example keyword prompts facilitated drawing out enough correspondence between Finland and South Korea among various sets of interview data. 

As such, the thematic analysis process did not consider the cultural and societal context of each country in mapping out the grounded similarities, but instead aimed at simplifying the structure of arts-health practices across all the study data from both Finland and South Korea. Furthermore, the intention was to investigate both the manifest and the latent content for the underlying interpretation.

## 4. Study Findings

A holistic perspective regarding participative service schemes was used in the analysis to assess the different stakeholders involved—older adults, practitioners, facilitators, and agencies—and their relationships in a diverse set of arts-health practices for older adults. As a result, the necessary components of designing arts-health practices for older adults’ creative well-being were identified by analyzing them from the planning stage to evaluation, and across the two countries and their cultural contexts. In fact, our findings showed parallel trends that exist within a comparative and cross-cultural contest in general.

The three central stages of organizing arts-health practices that were identified are *(1) identification (planning), (2) development and implementation,* and *(3) evaluation and dissemination.* Each of these three main categories contains two generic categories and a few additional sub-categories. Therefore, the findings of this study are examined here in these same three main categories. In addition, since Leadbeater’s [12] work—as discussed earlier—has provided a theoretical basis for this study, the findings of this study are also examined in relation to the approach taken by Leadbeater for formulating effective collective solutions that are developed based on bottom-up initiatives.

### 4.1. Stage 1: Identification (Planning)

The *identification (planning)* category encompasses all the themes that the interviewees shared in relation to their primary purpose or motivation in planning for, or participating in, arts-health practices. Firstly, the interviewees commonly noted that the perception of aging has changed dramatically, as older adults have become more productive and resourceful. In both countries, the majority of the interviewees mentioned that being considered “valuable” is crucial for older adults in becoming independent and preserving their sense of identity in later life. According to them, this issue did not just concern individuals but also affected society at large. For example, as part of their efforts to be respected and not to be a burden, they would, for instance, help take care of their grandchildren, or participate in volunteering work to promote the social values they have acquired through their aging and as part of their life experiences. 

Despite this, it was noted that outdated approaches to addressing the needs of older adults, irrespective of their circumstance—e.g., health, education, culture, wealth—have hindered their ability of our interviewees to generate positive outcomes that could become valuable assets to the development of a better society. The two generic categories identified as part of Stage 1—*cultural profiling* and *person-centered planning*—present the current challenges described here. The practitioners and facilitators interviewed in this study highlighted the need for the development of better approaches to quality care for older adults. Such approaches require the practitioners and facilitators to have the necessary proficiency in providing personalized creative stimuli to older adults. This kind of proficiency was noted to be directly correlated with the success of practitioners’ effective engagement, how well they interact with older adults to moderate appropriate topics and materials that reflect their interests, as well as how well they identify older adults’ changing values and goals as they age. According to the interviewees—especially practitioners and facilitators—this type of intimate interaction with older adults can be carried out through an in-depth profiling process based on older adults’ individual circumstances. Ultimately, the success of this kind of approach can determine the success of arts-health practices.

As our interviewed practitioners and facilitators in both countries observed, various social issues—such as the isolation of aging people, lack of community cohesion, and lack of supportive environments—threaten community values and its development. Therefore, arts-health practices are used as a means to resolve widespread existing obstacles for older adults, by encouraging their social engagement through everyday creativity. For instance, a few of the interviewees have developed arts-health practices for long-term local older adult community residents to encourage their equal access to social infrastructures and foster integration amongst all the community residents, both natives and immigrants. Such social engagements through creative interventions within artistic environments have helped to increase older adults’ sense of belonging, create mutual respect between residents, and enhance social networks toward community integration and development.

In terms of the theme of identification, the study findings have highlighted several emerging missions to providing effective quality care for older adults. These include *(1) generating positive outcomes through creative and dynamic engagements*, *(2) fostering social services and social engagement through a more inclusive and interactive approach*, and *(3) improving holistic care systems through collaborative partnerships within an interdisciplinary context*. These missions demonstrate the importance of an elaborate identification process as one of the critical strategic parts in initiating effective arts-health practices.

Taking on Leadbeater’s notion of “*intimate consultation*” as one of the invariable steps of participative services [12] (p. 57), intimate consultation can be used in arts-health practices as a way of figuring out the initial creative stimulus for older adults. Through the intimate consultation process, practitioners and facilitators can provide personalized support to older adults to uncover their needs, preferences, and aspirations by engaging in extended dialogues with them. This kind of sincere and thoughtful support can genuinely empower older adults—who have traditionally been overlooked in most cases—and help their role to be considered not as passive care subjects but as primary service users and decision-makers. Through this alternative perspective, older adults can become more engaged in arts-health practices and can take on an active role in establishing an equal and genuine partnership with other stakeholders involved in arts-health practices, rather than just receiving care as clients [15].

### 4.2. Stage 2: Development and Implementation

Partnership has become an increasingly important element in social policies, with a focus on more formal and often long-term partnerships involving diverse stakeholders [14]. In this regard, the *development and collaborative management* category includes the themes related to essential elements required for making arts-health practices more inclusive and interactive through genuine partnerships among all the stakeholders involved. The two generic categories identified in relation to Stage 2—*objective setting* and *compensatory strategy building (collaborative management)*—highlight the necessity of formulating novel strategies for modifying the prevalent existing structures—both theoretical and practical—for planning, coordinating, and communicating arts-health practices. Furthermore, codes included in the sub-category of Stage 2 relate to the fundamental components required for such novel strategies.

According to our interviewees, older adults are being increasingly recognized as active and responsible collaborators in their arts-health practices. In an aging society, older adults often challenge the delicate demands for quality care as main consumers of public services. Based on the experiences of our interviewees, the three primary objectives that the diverse participants of arts-health initiatives hope to achieve are *(1) positive well-being outcomes*, *(2) community involvement*, and *(3) increased accessibility*. In a similar vein to the main motivations behind many of the arts-health practices—as discussed in relation to Stage 1—a number of factors contribute to these objectives, including the complex needs of individuals in terms of their well-being and quality of later life, and the widespread societal concerns regarding the loss of community values and the social engagement of older adults. 

In terms of collaborative management of the development and implementation of arts-health practices for older adults, the study findings show that genuine cooperation toward the better execution of arts-health practices in an interdisciplinary setting can be established through active and open communication based on mutual trust, and having creative mindsets to achieve shared goals, while taking responsibility for different roles in a power-balanced environment. Such collaborative communication processes were assessed by our interviewees according to the way they reached a compromise when developing arts-health practices, to contribute to achieving common goals. 

The element of “*advocacy*”, as identified by Leadbeater [12] (p. 59), can be employed especially by practitioners and facilitators as a valuable tool for collaborative management in arts-health practices. According to Leadbeater [12], “professionals should act as advocates for users, helping them to navigate their way through the system” (p. 59). This means that, for example, practitioners and facilitators in arts-health practices need to advocate for older adults’ active participation by forming strong rapports with them and developing coherent care strategies with them. Such advocacy will enable older adults to feel more comfortable in maintaining intimate relationships with the practitioners and facilitators responsible for their care. Furthermore, in addition to advocacy for older adults, seeking support from many different domains—such as care ethics, philosophy, anthropology, and sociology—can be vital not only for the sustainable implementation of such practices through cross-disciplinary partnerships, but also for exploring the role of the arts in health, which has a vast and largely underutilized potential [65].

In this regard, our interviewed practitioners and facilitators shared their own experiences with utilizing various methods of advocacy. For example, they mentioned organizing different discussions with stakeholders and other people interested in arts-health practices, including internal and external professionals, institutional officials, and older adults. As opposed to what takes place in a more formal gathering, these discussions encouraged casual feedback and the exchange of advice throughout the course of arts-health practices. To ensure the effective implementation of each arts-health practice, in the discussion, participants continued monitoring each other’s performance extensively and adjusted the practice accordingly. There were also similar experiences among the interviewees in different contexts, facilitating hearing sessions with older adults to listen to their needs and wants and addressing common goals during their arts-health practices. In each case, although it was impossible to eliminate inherent power differences derived from funding issues or the level of expertise among different stakeholders and audiences, the effort to embrace diverse dialogues and requests—through advocacy—cultivated better collaborative decision-making processes and raised participation rates. In turn, such efforts improved the long-term sustainability of arts-health practices and achieved their ultimate goals in terms of social integration and community resilience.

### 4.3. Stage 3: Evaluation and Dissemination

The *evaluation and dissemination* category includes all the significant factors that need to be addressed for sustainable evaluation of arts-health practices in a more coordinated manner through partnerships. Sustainable evaluation of arts-health practices requires addressing a variety of challenges related to, for instance, the level of funding, capacity, knowledge, and skills, in addition to ethical issues and governance concerns [66]. It is, therefore, not surprising that art practitioners and organizations struggle to negotiate the minefield of evaluation in arts-health practices [66,67,68,69,70,71,72].

Our study highlighted these different challenges and confirmed the lack of creative and efficient methodologies for evaluation of arts-health practices when measuring both their quantitative and qualitative outcomes. It was observed many times that pursuing diverging outcomes by different stakeholders deteriorated the quality of evaluation under power imbalance and unequal circumstances. Many of our interviewees noted that the conflicting interests due to external factors related to funding, resources, employment, and infrastructure often affect the assessment and interpretation of the outcomes of arts-health practices. This, in turn, impedes the continuation of such practices, and prevents different stakeholders from making sense of those practices and learning from them individually or collectively.

Several of the interviewed practitioners and facilitators also expressed their frustration with the lack of coordination in dealing with interdisciplinary issues and the undervaluation of evidence they have observed in real-world contexts, instead of focusing on evaluation as part of academic research that scholars have theorized beforehand. These observations suggest that there is a need for critical reflections on responsible actions, beginning with the idea of “*governmentality*”, where empowerment becomes a factor in a self-regulated and collaborative environment. Such critical reflections result in a number of key questions in terms of who defines an “effect” and how it is examined from the perspective of people who feel valued rather than people who add value. It also becomes important to understand what dominances are hidden under evaluation due to taking control of different responsibilities, and how these dominances govern certain rules and rationales invisibly in an interdisciplinary context. These questions then lead to finding out how existing barriers in an interdisciplinary environment can be overcome, to lead to making substantial improvements in dealing with these issues systematically. 

The two generic categories defined in relation to Stage 3—*professional development (competencies)* and *sustainability of practices*—related to the need for sharing visions and resources among stakeholders to re-establish responsibility and systematic operation in an interdisciplinary context. In terms of *professional development (competencies)*, our study shows that it is necessary to develop a multi-framed evaluation structure that supports discussions for further actions in arts-health practices. Such a multi-framed evaluation structure can make stakeholders carefully reflect on their multiple views of situations and goals throughout the entire process of arts-health practices [14]. For this evaluative structure to work, practitioners and facilitators need to develop their unique and competent skills in “facilitation, trust-building, reflecting, negotiating, resource-finding, interpretation, and conflict-management” [14] (p. 10), in order to carry out the evaluation and dissemination processes thoroughly in a systematic manner. 

In relation to *sustainability of practices*, the interviewees often stated their wishes that their experiences would affect the wider community rather than remaining within them. For example, some of the interviewed older adults wished that their knowledge from previous experiences would be utilized as mentorship ideas, or for making improvements to future arts-health practices. Furthermore, some older adults wanted to take charge of their programs and implement them independently in different forms and settings in their own ways. Practitioners and facilitators, on the other hand, mentioned seeking out additional opportunities, such as round table meetings, discussions, and workshops with other professionals. They felt that such opportunities would enable them to exchange ideas and opinions about how to broaden the scope of their arts-health practices through exploration of further joint working opportunities.

Overall, the results of this study show that equal partnerships, along with a sense of dignity, can promote barrier-free interactions and make all the stakeholders involved more productive at delivering better care services for older adults in the long term. Although the availability and sustainability of resources directly affect different stakeholders’ ability to plan and implement activities [73], the ultimate goal of arts-health practices should be the catalyzation of communal efforts to align goals and agendas across sectors, to preserve the legitimacy of such practices.

## 5. Models of Arts-Health Practices

As noted earlier, the aim of this study has been to investigate different perspectives of all the stakeholders involved in arts-health practices for older adults, and to map out the relationships between these stakeholders as collaborators, while also highlighting the importance of older adults themselves in becoming active partners in arts-health practices that lead to improving their late-life creative well-being. Based on the findings of the study presented above, two complementary models are proposed here to support the development of an interdisciplinary environment that aims to bridge unsettled relationships and distribute existing responsibilities among different stakeholders from various fields involved in arts-health practices. The two proposed models are a *concept map* and a *cyclic conceptual framework*.

### 5.1. Concept Map

**Figure 1:**
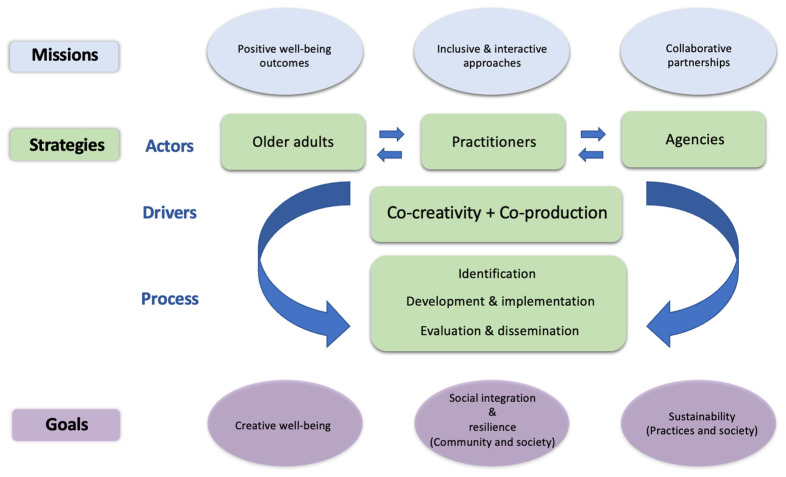
The proposed concept map for arts-health practices.

Figure 1 shows an overview of the proposed *concept map* that presents the *missions*, *strategies*, and *goals* of collaborative arts-health practices for older adults, as well as the *actors*, *drivers*, and *processes* involved in them. This model aims to map out the integrated operations among different stakeholders and to bring together the theoretical and practical aspects of planning, coordinating, and communicating in arts-health practices.

As examined earlier, the core concept of each central stage of arts-health practices intertwines with the key themes of *missions*, *strategies*, and *goals* required for the operation of arts-health practices. Therefore, the proposed concept map is organized according to these three themes. The *strategies* theme is further divided into three elements of *actors*, *drivers*, and *process* that are linked to achieve the common goals of arts-health practices and support their effectiveness in a systematic manner. These three elements can be considered as methodological tools that are used to support the execution of different strategies—through active involvement of different actors (stakeholders)—and developmental milestones within the service delivery process. 

In terms of the driving forces in arts-health practices and service delivery for older adults, it is important to highlight the notion of *co-creativity* and *co-production*. As noted earlier, co-production and co-research with older people have gained importance in recent years due to increasingly aging populations and the demand to improve services and policies that are more inclusive and responsive [6]. Therefore, the concept map presented here adopts co-creativity and co-production as driving forces for shaping arts-health services, encompassing the entire process in which users—i.e., older adults—are expected to take an active and responsible role in their service delivery [12]. 

On the question of the users’ responsibility in the delivery of arts-health practices, the co-production approach is also closely related to the fields of social and community work, as part of efforts to provide “a renewed commitment” [6] (p. 52) toward creating “more equitable, inclusive, and responsive services” [14] (p. 14). Such a renewed commitment can increase the successful implementation of arts-health practices when the target user group—older adults in this case—is directly involved in the decision-making process in a more inclusive way, and by doing so, can improve the planning, management, and running of services—i.e., arts-health practices. As part of this, it is important to consider the late-life creativity of older adults as an asset in arts-health practices, which older adults can utilize to strengthen their capacity and influence their contributions to the decision-making process.

Another significant aspect that needs to be examined here in terms of co-creativity and co-production is the generation of meaningful dialogues between service users and their providers. A creative environment requires a range of different dynamics within the co-production process to enable stakeholders to actively exchange their knowledge and build robust partnerships based on mutual trust, and by resolving power relationships between them. According to Zeilig et al. [74], co-creativity is not currently defined as a single entity, but rather as “a number of key features including centrally: a focus on shared process, the absence of a single author or outcome (and instead the idea of shared ownership), inclusivity, reciprocity, and relationality” (p. 138). Similarly, Tischler [75] notes that “emergent findings indicate that this approach [co-creativity] is found to benefit all involved and that it promotes transdisciplinary processes, i.e., those that move beyond disciplinary boundaries, enacting change in practices” (p. 87). Based on these, it is clear that arts-health practices can leverage the strength of co-creativity to support interdisciplinary partnerships between the consumers of such services—i.e., older adults—and the providers of those services, which then converge into co-production.

### 5.2. Cyclic Conceptual Framework

As pointed out by Tan [76], despite the fact that arts-health practitioners and facilitators have been increasingly focusing on developing facilitation techniques to improve their participants’ experiences and their well-being [72,77,78,79,80], there are not many conceptual models that they can utilize to guide their work. Therefore, Tan [76] reviews the few relevant existing models and notes that most practitioners and facilitators are still clarifying the key components of their arts-health practices, with the hope of ensuring that their participants would have satisfying and enriching experiences. 

**Figure 2:**
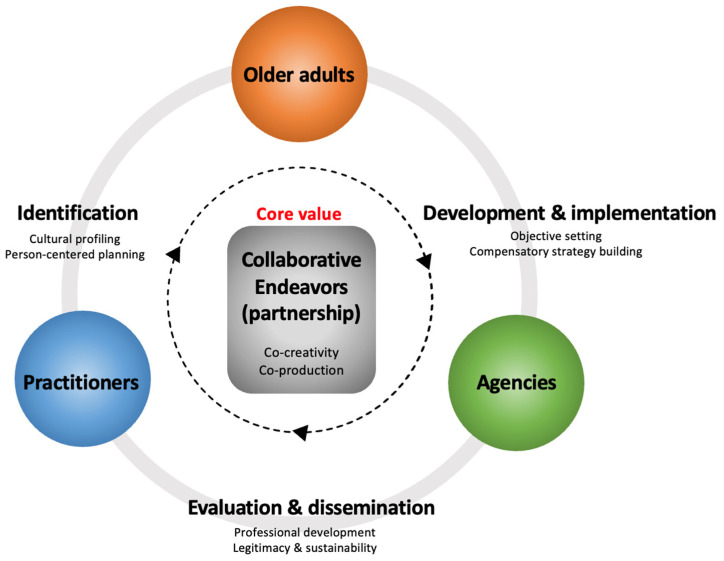
The proposed cyclic conceptual framework for facilitating arts-health practices.

This lack of conceptual models has indeed been a motivation behind conducting the study presented here. Our overarching aim has been to structure the dynamics of interactions between all the stakeholders in arts-health practices and present them in the form of a conceptual framework to support their operations. Figure 2 presents the proposed cyclic conceptual framework for facilitating arts-health practices. This framework also takes into account different constraints specific to the complex circumstances of cooperative operations in arts-health practices.

The framework emphasizes the need to identify ways of increasing collaborative interplay and addressing constraints specifically associated with the complex context of the dynamics of interactions among different stakeholders. The key stakeholders in arts-health practices—*older adults, practitioners,* and *agencies*—are therefore shown as the main operators in a circular operation model, which includes the major strategic points from the generic categories identified in our study—as shown in Table 2 and discussed earlier. The major strategic points in this conceptual framework are the essential methods in each of the three stages of the arts-health practices, which allow the whole process to be adjusted in an iterative manner. As such, the inner circle of this model with arrows signifies how arts-health practices tend to be applied iteratively along several strategic points. 

The framework also centralizes the notion of *collaborative endeavors (partnership)* among the key stakeholders to highlight the core value of the whole operation. As discussed in relation to the conceptual map, the core value of the arts-health practices—*co-creativity* and *co-production*—can be achieved through *collaborative endeavors (partnership)* among the key stakeholders. In fact, a co-production environment demands a strong and complex support mechanism and supervision provisions for practitioners—from peers or agencies—to reduce their high levels of stress and discomfort [14]. This is because a co-production environment requires those involved in them to cope with uncertainty, ambiguity, and challenges of different kinds [14]. Therefore, the framework requires the co-production environment to operate through dynamics of interaction based on collaborative endeavors, such that it would systematically provide for both peer support and multi-layered supervision. In other words, the framework proposes that the key stakeholders involved in arts-health practices should reduce the level of uncertainty linked to the nature of co-production, while also cultivating a long-term impact with each other through their robust partnerships, by benefiting from their collaborative endeavors.

As Fortier and Coulter [73] point out, the legitimacy of arts-health practices depends on the level of cross-sectoral relationships, whether weak or strong. Such legitimacy should be perceived differently in different sectors, and under different conditions or situations. This means that the core value of the proposed conceptual framework—*collaborative endeavors (partnership)*—aims to empower the key stakeholders in recognizing their responsibilities and contributions to one another toward creating a strong alignment. This form of strongly aligned cross-sectoral interests would strengthen relationships, thus resulting in a greater sense of legitimacy, easier access to resources, and a larger capacity to act [73]. Hence, it is expected that such alignment will eventually enable the key stakeholders in arts-health practices to expand the limits of their working boundaries, which would otherwise hinder their ability to implement their plans more effectively or in a different manner.

## 6. Conclusions

In this article, we have presented a study investigating empirical perspectives of different stakeholders—older adults, facilitators, practitioners, and agencies—involved in a wide range of arts-health practices for older adults. This study has looked at the relationships between those stakeholders, and their responsibilities and roles toward co-creativity and co-production in arts-health practices. As part of this, the necessary elements required for developing arts-health practices for older adults’ creative well-being have been identified by analyzing such practices from their planning stage to their evaluation across the cultural contexts of Finland and South Korea. 

The results of this study have demonstrated the need for developing methods of quality care in a systematic way to catalyze communal efforts and the delivery of long-term effects. In particular, methods of creating collaborative partnerships among multiple stakeholders in arts-health practices are seen as much needed, especially those emphasizing the involvement of older adults as co-producers and co-decision-makers, and utilizing their late-life creativity and life-long experiences. 

To assist the development of future methods of collaborative partnerships, a concept map and a cyclic conceptual framework for arts-health practices have been proposed based on the findings of this study. These two models emphasize the role of co-creativity and co-production as powerful strategic tools and drivers in arts-health practices for generating meaningful dialogues between different stakeholders, and toward the delivery of better care services for older adults. 

Finally, it is important to note that, as of yet, there have been no empirical studies to support the notion of co-production in arts-health practices, along with the ethical and methodological issues around it [19]. Despite this, our study suggests—based on the perspectives of different stakeholders involved—the positive value of collaborative partnerships generated through co-creation and co-production in arts-health practices that cultivate long-term social impact. In addition, older adults’ continued participation in such arts-health practices—as key decision-makers—can lead to improved self-efficacy in dealing with countless challenges they face every day by utilizing their life-long experiences, knowledge, and late-life creativity.

## Figures and Tables

**Table 1:** Summary overview of sources of data used for this study.

Forms of Data	Set	Participants	Site	Group Description	Primary Purpose
**Individual interviews**	I1	9 older adults (aged 58–86)	Finland	− 6 women living in a housing community in the urban Helsinki region, who engage in various creative group activities (e.g., photography, crafts, creative writing). − 3 women who have participated in arts-based and other community-based societal activities in creative ways (e.g., painting, crafts, environmental activism, volunteering).	− To understand older adults’ personal aging experiences. − To identify the needs of older adults in terms of arts-health practices for well-being (physically, emotionally, and socially).
	I2	15 practitioners/ facilitators	Finland and South Korea	− 15 practitioners/facilitators actively working with/for older adults in public and private sectors by using arts as a means to interact with them (e.g., drama, drawing, painting, crafts, performance).	− To understand arts-health practices in different contexts. − To understand the mission and goals of arts-health practices and the value of creative engagements for older adults.
**Focus groups**	F1	9 older adults (aged 58–76)	Finland	− 9 women involved in several artistic projects (e.g., performance, textile work) in collaboration with local community artists.	− To obtain feedback about participation in arts-health practices, by sharing experiences and reflection.
	F2	10 older adults (aged 68–82)	South Korea	− 10 women who have participated in a drama activity using their life stories.	
**Final** **reports**	R1	4 practitioners/ facilitators	Finland and South Korea	− 4 reports of both qualitative and quantitative evaluations of specific practices conducted with/for older adults.	− To gain a comprehensive understanding of specific arts-health practices. − To review arts-health practice procedure and access their resources (quantitative and qualitative materials and visuals).

**Table 2:** A summary of the categories identified in the study data using the inductive content analysis method.

Main Categories (Central Stages)	Generic Categories	Sub-Categories
**Stage 1:** **Identification** **(planning)**	− Cultural profiling (problem identification); − Person-centered planning.	− Understanding generational characteristics; − Generating continued interest and participation.
**Stage 2:** **Development** **and implementation**	− Objective setting; − Compensatory strategy building (collaborative management).	− Proper tools, materials, and medium; − Promoting well-being (mental and physical); − Accessibility and openness; − Diversity and flexibility; − Peer-group support.
**Stage 3:** **Evaluation and** **dissemination**	− Professional development (competencies); − Sustainability of practices.	− Localization and decentralization; − Quantity vs. quality, and process vs. result; − Funding capacity; − Stability of employment; − Infrastructure development.

## Data Availability

The authors do not have permission to share their study data.
